# The Impact of Cropland Abandonment of Post-Soviet Countries on the Terrestrial Carbon Cycle Based on Optimizing the Cropland Distribution Map

**DOI:** 10.3390/biology11050620

**Published:** 2022-04-19

**Authors:** Shengjie Zhou, Tiexi Chen, Ning Zeng, Qixiang Cai, Fang Zhao, Pengfei Han, Qingyun Yan

**Affiliations:** 1 Collaborative Innovation Center on Forecast and Evaluation of Meteorological Disaster, School of Geographical Sciences, Nanjing University of Information Science & Technology, Nanjing 210044, China; 20181210015@nuist.edu.cn; 2 School of Geographical Sciences, Qinghai Normal University, Xining 810016, China; 3 State Key Laboratory of Numerical Modeling for Atmospheric Sciences and Geophysical Fluid Dynamics, Institute of Atmospheric Physics, Chinese Academy of Sciences, Beijing 100020, China; zeng@lasg.iap.ac.cn (N.Z.); pfhan@mail.iap.ac.cn (P.H.); 4 School of Geographic Sciences, East China Normal University, Shanghai 200241, China; fzhao@geo.ecnu.edu.cn; 5 School of Remote Sensing and Geomatics Engineering, Geographical Sciences, Nanjing University of Information Science & Technology, Nanjing 210044, China; 003257@nuist.edu.cn

**Keywords:** dynamic global vegetation model, Post-Soviet cropland abandonment, carbon cycle

## Abstract

**Simple Summary:**

After the collapse of the Soviet Union, changes in the agricultural structure led to widespread abandonment of cropland and natural vegetation restoration in Russia, Ukraine, and Belarus. In consequence, corresponding changes in the terrestrial carbon cycle need to be quantified. We simulated this process using a dynamic vegetation model, and found that the conversion of cropland to natural vegetation generally formed a significant carbon sink at 0.99 GtC; the growth of the vegetation carbon pool, especially, was significantly higher than that of soil carbon pool.

**Abstract:**

Land use and cover changes (LUCC) have a fundamental impact on the terrestrial carbon cycle. The abandonment of cropland as a result of the collapse of the Soviet Union offers a typical case of the conversion from cropland to natural vegetation, which could have a significant effect on the terrestrial carbon cycle. Due to the inaccuracy of LUCC records, the corresponding impact on the terrestrial carbon cycle has not been well quantified. In this study, we estimated the carbon flux using the Vegetation-Global-Atmosphere-Soil (VEGAS) model over the region of Russia, Belarus and Ukraine during 1990–2017. We first optimized the LUCC input data by adjusting the Food and Agriculture Organization (FAO) data by Russian statistical data and redistributing the spatiotemporal input data from the Historical Database of the Global Environment (HYDE) to the original model. Between 1990 and 2017, the area of cropland abandonment was estimated to be 36.82 Mha, compared to 11.67 Mha estimated by FAO. At the same time, the carbon uptake from the atmosphere to the biosphere was 9.23 GtC (vs fixed cropland 8.24 and HYDE 8.25 GtC) during 1990–2017, which means by optimizing the cropland distribution data, the total carbon absorption during the abandonment process increased by 0.99 GtC. Meanwhile, the growth of the vegetation carbon pool was significantly higher than that of the soil carbon pool. Therefore, we further highlight the importance of accurate cropland distribution data in terrestrial carbon cycle simulation.

## 1. Introduction

The terrestrial ecosystem carbon cycle plays an important role in the global carbon cycle, and is the main component in the research of atmospheric CO_2_ concentration and climate change, as well as the interaction between the atmosphere and the biosphere [1]. Land use and cover change (LUCC) has a direct and fundamental contribution to the carbon source and sink process of the terrestrial ecosystem [2,3,4]. The shift from one type of land cover to another is often accompanied by a large amount of carbon exchanges [4]. The expansion and abandonment of cropland are one of the typical processes of LUCC. With the increasing demand for food and feed, cropland as a whole showed great growth in the 20th century [5,6,7]. At the same time, because of social policies, croplands were reduced in some areas. Typical cases are the abandonment of cropland due to the collapse of the Soviet Union [8] and the implementation of ecological projects in China to return the croplands to forest and grasslands [9].

Before and after the collapse of the Soviet Union, great changes took place in cropland in eastern Europe as a result of the change of the social economic system and land management policy [10]. During the Soviet period, high-intensity reclamation was induced due to agricultural collectivization and intensification of production [11,12]. Due to the change of social policy and economic structure, the shrink of agricultural scale resulted in the phenomenon of large-scale cropland abonnement [11,12,13,14]. The amount and distribution of the cropland are still not well documented, which leads to large uncertainties in its impact on terrestrial carbon cycle [15].

The abandonment and re-cultivation of cropland in this area make the whole process very complicated. For instance, forests are regenerating due to the abandonment of marginal cropland [16,17,18], which could help mitigate the impact of climate change by increasing carbon sequestration [19,20]. The environmental and economic costs of reclaiming these lands for crops could be significant. Although some of this abandoned cropland has been replanted since 2000, partly because of growing support for agriculture and rising commodity prices, the majority of them remains abandoned and is gradually returning to forests [21,22]. 

Global dynamic vegetation models (DGVMs) are effective tools in the investigation of the impact of land use cover change on the carbon cycle of terrestrial ecosystems [15,23]. Meanwhile, the quality of model-input LUCC data plays an essential role in simulation. In recent years, several projects have led to significant progress in the reconstruction of the past environment and, in particular, the global land cover of the past 300 years [24]. For instance, LUCC data from the Historical Database of the Global Environment (HYDE) established by the Netherlands Environmental Assessment Agency is widely used [25,26], and the agricultural statistics of the Food and Agriculture Organization (FAO) is one of the main sources of HYDE data [15].

Local-scale LUCC data usually could be further optimized compared to the global covered dataset. Here we noticed a potential problem involved with the cropland abandonment of the Soviet Union. Data from FAO are clearly problematic, as they fail to capture the total area of abandoned croplands, leading to an overestimation of current arable land area. Studies have shown that Russia has the highest abandonment rate among all the Soviet Union countries [15], and the FAO estimates the country’s arable land from 1992 to 2008, mainly based on the official report and inquiry or FAO’s own estimate; meanwhile, extrapolating 2009 to the current year using the value in 2008 is also one of the indications that the FAO underrates dynamic change in the Russian stock of regional agricultural land. This misestimate will be brought into the HYDE data that was produced mainly based on FAO records, and thus further affect the results of dynamic vegetation models driven by it. 

Therefore, the basic objective of this paper is to estimate the carbon pool changes induced by cropland abandonment using a global dynamic vegetation model with difference LUCC scenarios, including using optimized croplands, default HYDE records and fixed croplands.

## 2. Materials and Methods

### 2.1. Study Area

**Figure 1:**
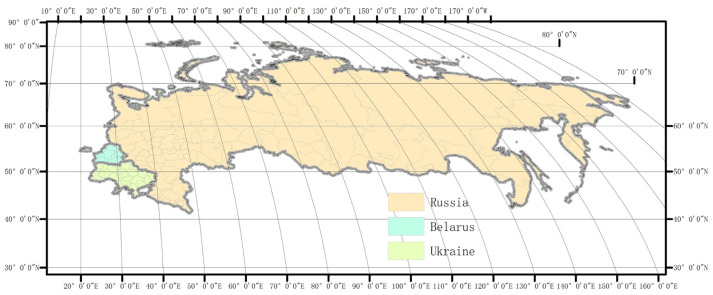
Geographical location of the study area.

According to FAO statistics, the Soviet Union’s croplands accounted for 10.44% of its land area (or 233.88 Mha based on the total land area of 2240.22 Mha) in 1991, within which Russia had the largest croplands (57.17%). Previous studies also demonstrated that Russia contributed the most in cropland abandonment [15,27]. European Russia is the major agricultural region connected to the agricultural regions of Ukraine and Belarus. This region is also one of the largest mollisol distribution areas in the world. Therefore, in the study, we choose these three countries as the study area (Figure 1), which is basically consistent with the study area selected in previous studies. Given that the Soviet Union had already seen a decline in cropland prior to its break-up, these three countries probably accounted for a higher proportion of the total cropland of all Soviet states in 1992. 

### 2.2. Land Use Data

Land use includes two parts: the default data and optimized land use data that we created. The 1/12-degree gridded land use data from HYDE (v3.2) is selected as the default, which was developed by the Netherlands Environmental Assessment Agency [28]. The temporal resolutions of HYDE are every ten years since 1700 and every one year after 2000. The HYDE dataset contains three land use types, including cropland, pasture or grazing land, and urban. Country-level statistics of cropland used to drive the HYDE data were from FAO cropland records. As illustrated in the definitions and standards used in FAOSTAT [29], FAO cropland records were generated mainly based on several methods: data reported in country official publications, official data reported in FAO questionnaires from countries, FAO estimate, and manual estimation. 

Then, we conducted a calibration method of cropland area in the RUB region. Clarifying the concept of cropland is the prerequisite because these datasets would have subtle differences in the definition of cropland. The FAO defines cropland as the arable land with the addition of permanent crops. However, in the definition of Russian official publication, arable land is land systematically cultivated used for sowing of agricultural crops, including sowing of perennial grasses and complete fallows. The actual sowing area is theoretically accurate for model input because croplands are always cultivated in the simulation without fallow. We also noticed that due to the lack of annual updates, FAO-based cropland in Russia has little interannual variations during 2008–2017, which is far from the reality. Therefore, firstly, we recalibrated the total cropland area for model simulation.

Cropland records from the Federal State Statistics Service of Russia (Federal State Statistics Service (ROSSTAT)) were treated as the reference of cropland variations, which could reflect real interannual changes in croplands. The reliability of cropland data from ROSSTAT in land cover change studies has been previously recommended and validated [15]. 

Spin-up and historical simulations prior to 1990 still require the use of HYDE data. To avoid the discontinuity of LUCC data with a breakpoint in 1990, the ROSSTAT records could not be used to replace the HYDE data directly. The objective of this study is to investigate the impact of cropland change on the terrestrial carbon cycle. We used the changes in ROSSAT on HYDE rather than using ROSSAT values directly. Therefore, the FAO records were adjusted using the ROSSTAT records after the collapse of the Soviet Union. The optimized cropland in Russia (crop_rus_opt) since 1991 was calculated using Equation (1) as:

crop_rus_opt (i) = crop_rus_FAO (1990) + crop_ROSSTAT(i) − crop_ROSSTAT (1990)(1)

where i = 1991–2017, crop_rus_opt is the optimized cropland in Russia during 1991–2017 and crop_rus_FAO (1990) is FAO based cropland area in 1990, which keeps the data continuous without a break in 1990. This is required for the operation of the model, because the simulation starting point of the model is much earlier than 1990, and HYDE data were always used before that.

Meanwhile, previous studies have demonstrated that the abandonment rate of Ukraine (8%, between 1990–2008) and Belarus (9%, between 1990–2003) are much lower than that of Russia [15,27]. Therefore, we used the FAO records from these two countries directly. The total amount of cropland for the three countries could be re-established by multiplying crop_adrate with FAO records during 1990–2017. Because gridded records are needed as the input for the models, optimized gridded cropland areas were calculated by adjusting the HYDE cropland proportion of each grid using the crop_adrate values during 1990–2017 over the three countries as:

crop_opt(j) = HYDE(j) ∗ crop_adrate(j), j = 1990–2017(2)

### 2.3. Dynamic Vegetation Model

Several DGVMs have been developed in the last three decades which play an essential role in terrestrial carbon cycle research [30]. Here we selected a sophisticated model, called the Vegetation-Global-Atmosphere-Soil (VEGAS) model, to simulate the terrestrial carbon cycle [31]. This model was also involved in the TRENDY project to estimate the global carbon budget [32]. A benchmark application of the VEGAS model is that agricultural modernization promotes cropland productivity, which can further explain the increasing trend of the atmospheric CO_2_ concentration seasonal amplitude [30].

The VEGAS model includes 5 PFTs (plant function types): broadleaf tree, needleleaf tree, cold grass, warm grass, and cropland. The different photosynthetic pathways are distinguished for C3 (the first three PFTs above) and C4 (warm grass) plants. Photosynthesis uses a Jarvis-type formula with dependence on soil moisture, temperature, CO_2_, and light, modified by a co-limitation function following Collatz et al. [33] which causes a more gradual response to any change in a single factor. Photosynthesis also interacts with evapotranspiration in the physical land-surface model. The light dependence is not only a function of photosynthetically active radiation, but also a function of LAI and vegetation height structure. 

Accompanying the vegetation dynamics is the full terrestrial carbon cycle, starting from photosynthetic carbon assimilation in the leaves and the allocation of this carbon into five vegetation carbon pools: leaf, fine root, coarse root, sapwood, and heartwood. After accounting for respiration, these five vegetation carbon pools may die and turn over into two litter pools: metabolic (leaf and fine root) and structural (coarse root and wood). The decomposers (bacteria, fungi, insects, animals, and humans) are represented by a single carbon pool that works on the litter carbon, partly respired as heterotrophic respiration and partly as decomposed organic matter which cascades into three soil pools: fast, intermediate, and slow. Thus, there are a total of 6 ‘soil’ carbon pools, including litterfall and decomposer. Temperature- and moisture-dependent decomposition of these carbon pools returns carbon back into the atmosphere, thus closing the terrestrial carbon cycle.

Simulations were made using three LUCC scenarios. (1) Using 1990 cropland as a constant reference during 1990–2017. (2) Using HYDE records as the default scenario. (3) Using optimized croplands as the real values. The differences of the carbon pools between Scenario 3 and Scenario 1 are treated as the impact of cropland abandonment. The differences of the carbon pools between Scenario 3 and Scenario 2 are treated as the simulation improvements with optimized cropland distribution.

## 3. Results

### 3.1. Optimized Cropland Area and Distribution

**Figure 2:**
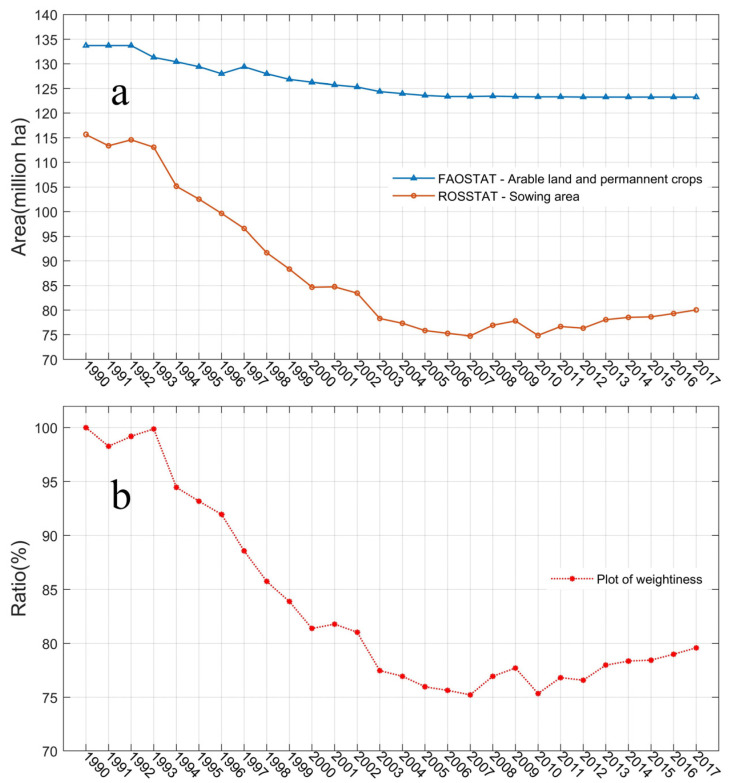
Russia interannual cropland extension and adjusted rate used in this study. (**a**) annual cropland area indicated by FAO arable land (blue) and the ROSSTAT sown area during 1990–2017 (red). (**b**) Copland adjusted rate which was applied to generate the optimized cropland using Equation (2) (see methods section).

As illustrated in Figure 2, total cropland area of Russia based on FAO records only showed a slight decreasing trend during 1992–2017, which is obviously problematic. In contrast, the sown area has decreased sharply since 1992, which reaches the bottom at about 2007 using the ROSSTAT records (Figure 2a). The sown area in Russia has dropped from about 114.59 Mha to 80.05 Mha from 1992 to 2017, with the minimum record of 74.76 Mha in 2007. The cropland area indicated by FAO data changes from 133.71 Mha to 123.25 Mha during this period, which could not capture the basic procedure of cropland abandonment.

The adjusted rate using Equation (2) is illustrated in Figure 2b, which was mainly affected by the changes of sown area of Russia (Figure 2a). The lowest adjusted rate, 0.74, appeared in the year 2007, and during 2003–2017, all the values are below 0.8, which reflect a large correction on the original data.

**Figure 3:**
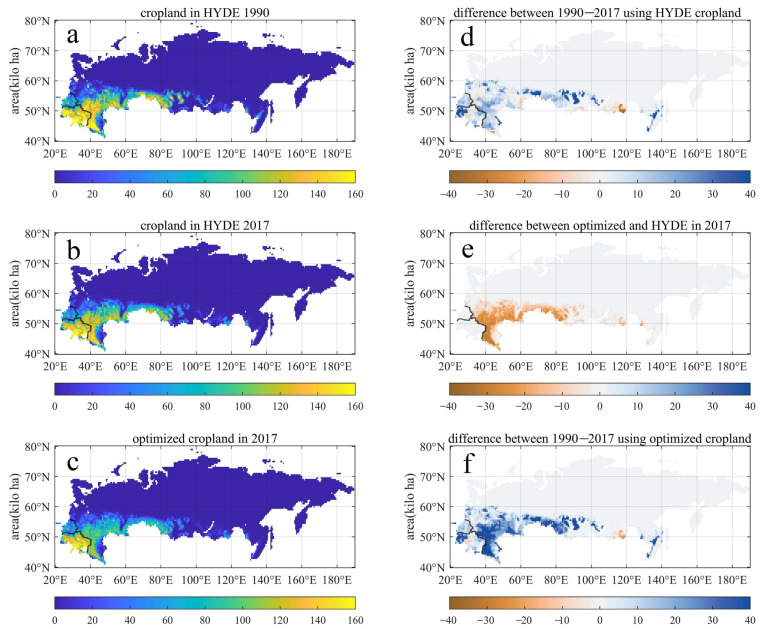
Default and optimized cropland distribution used in this study. (**a**) the cropland distribution at the beginning (1990) of our study period. (**b**) the default HYDE cropland distribution in 2017. (**c**) optimized cropland distribution in 2017. (**d**) the differences between optimized and default cropland in 2017. (**e**) the difference in cropland in 1990 compared to 2017 using the HYDE records. (**f**) the difference in cropland in 1990 compared to 2017 using the optimized records.

Optimized gridded cropland distribution records are demonstrated in Figure 3. According to HYDE data, the croplands in the RUB region are mainly distributed in the central and southern plains of Eastern Europe, and a small part in southern Siberia and the Far East. As mentioned above, in this work the calibration process does not change the distribution characteristics of HYDE, but significantly reduces the areas of cropland. 

The default croplands distribution using HYDE data exhibited little observable changes from 1990 to 2017 (Figure 3a,b), which is unreasonable as illustrated by previous studies [8,15]. The optimized data shows that the cropping intensities of the main cropland distribution areas have dropped significantly in 2017 (Figure 3c). The quantification of the differences between optimized and default cropland in 2017 is illustrated in Figure 3d which exhibits an overall decline. In consequence, the difference in cropland in 1990 compared to 2017 using both HYDE and optimized records have large biases in describing the decline in croplands caused by the abandonment (Figure 3e,f).

### 3.2. Land Cover Conversion between Croplands and Natural Vegetation

**Figure 4:**
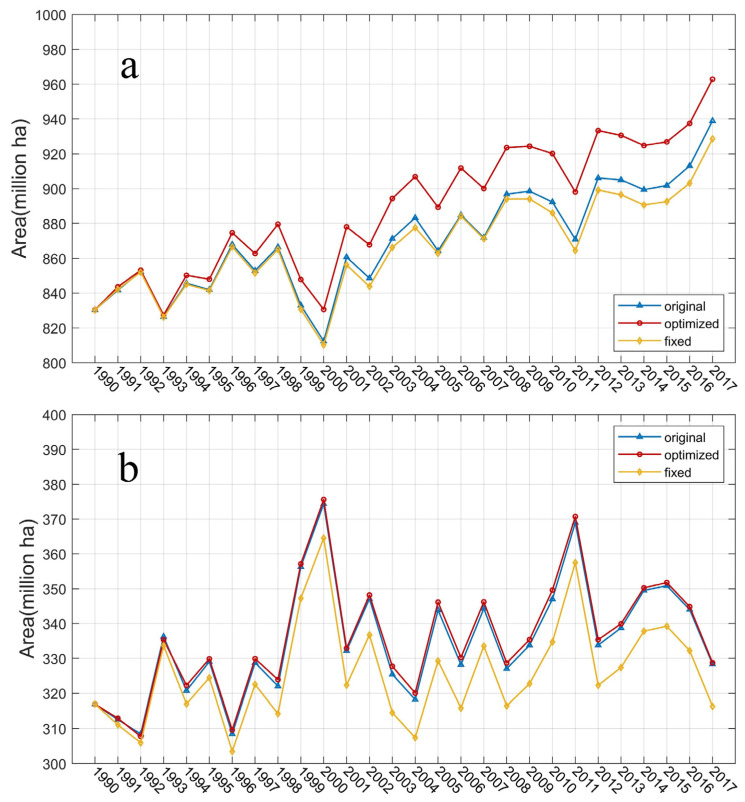
The time series of natural vegetation extensions simulated by VEGAS. (**a**) annual forest areas. (**b**) annual grassland areas. The blue line indicates the results with original cropland from HYDE. The red line is optimized cropland and the yellow line is fixed cropland which never changed since 1990.

**Figure 5:**
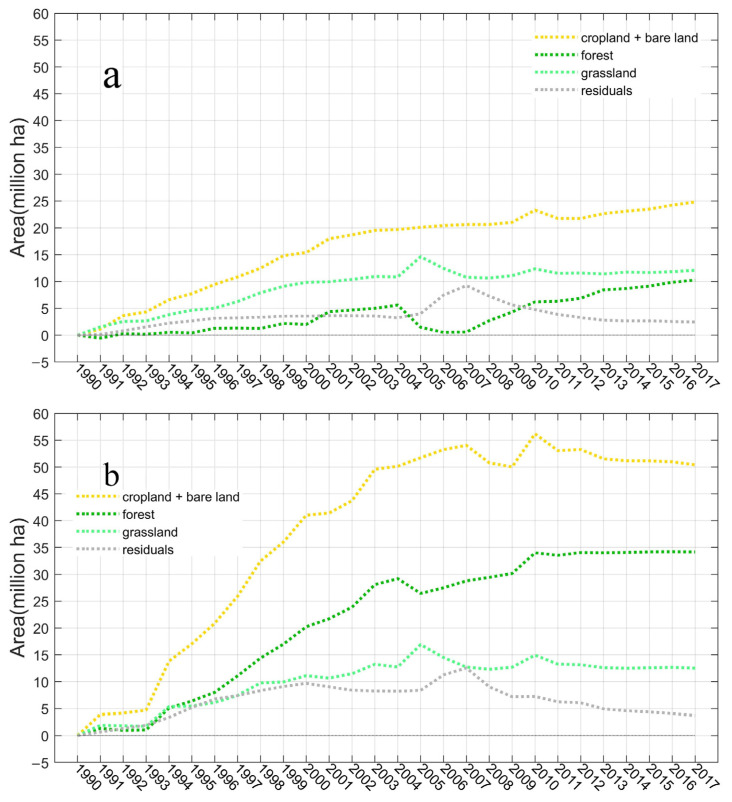
The time series of cropland abandonment and natural vegetation extensions caused by cropland abandonment. (**a**) simulations driven by the default HYDE cropland. (**b**) simulations driven by the optimized cropland. The yellow line indicates the cropland with attaching bare land reduction area compared to 1990. The dark green line is forest extension. The light green line is grassland extension and the grey line exhibits the residuals between cropland reduction and natural vegetation growth on abandoned cropland.

The vegetation functional type results simulated by optimized cropland show that under the influence of climatic conditions, most of the reduced cropland will be quickly transferred to grassland and forests. Here, we conducted a set of controlled experiments with three situations of cropland distribution using the default HYDE cropland, the optimized cropland, and the scenario that cropland never changes since 1990, respectively. In this way, we can confirm the contributions of the cropland abandonment process to natural vegetation expansion and the variation of the carbon pool. From 1990 to 2017, forest area increased by 132.46 Mha (Figure 4a) in total (including both the coniferous and broadleaved forest types), of which about 34.27 Mha was transferred from cropland (Figure 5b). In the same period, the grasslands increased by 11.83 Mha (including cold grass and warm grass); about 12.45 Mha was from cropland (simulated grassland area has interannual fluctuation, and results using fixed cropland show that grassland reduced in 2017 compared with that in 1990). Therefore, in total, about 46.72 Mha of natural vegetation were extended until 2017 in the cropland optimized simulation. On the other hand, the results simulated by the original HYDE cropland data show the forest area increased by 108.56 Mha with cropland abandonment contributing 10.27 Mha, and the grassland area increased by 11.43 Mha, of which 12.10 Mha was from croplands (Figure 4b).

### 3.3. Vegetation and Soil Carbon Pools

**Figure 6:**
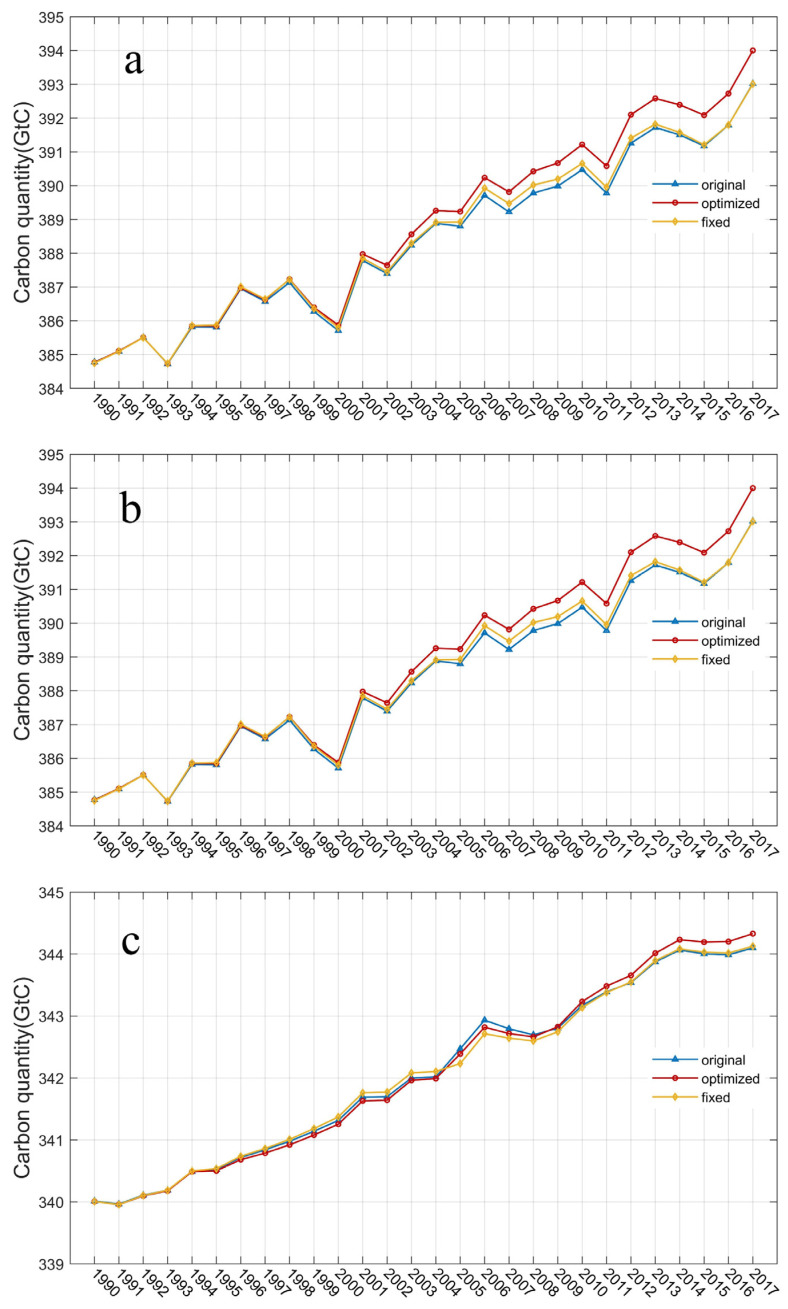
Changes in the carbon pool of RUB region using HYDE, optimized, and fixed cropland. (**a**) the total biosphere carbon pool. (**b**) the vegetation carbon pool. (**c**) the soil carbon pool. The blue line indicates the results with original cropland from HYDE. The red line is optimized cropland and the yellow line is fixed cropland which never changed since 1990.

Vegetation and soil carbon pools changed significantly because of the LUCC and climate change. As illustrated in Figure 6a, the total biosphere carbon pool was about 384.77 GtC in 1990. In 2017, the end of the study period, the total carbon pool simulated by fixed, HYDE, and optimized croplands are 393.01, 393.02, and 394.00 GtC, respectively. Therefore, using fixed cropland and default HYDE cropland as input, the total biosphere carbon sink in the RUB region increased by 8.24 GtC and 8.25 GtC during 1990–2017, respectively. In the optimized simulation of the same period, the total biosphere carbon sink increased by 9.23 GtC, which is equivalent to a net carbon sink of 0.34 GtC per year. Therefore, using optimized cropland areas, an additional 0.98 GtC (or 0.99 GtC) in carbon sink was achieved compared with the default HYDE (or with fixed croplands) records. Therefore, croplands changes contribute a carbon sink of about 0.04 GtC per year. The vegetation carbon pool and soil carbon pool were further analyzed separately (Figure 6b,c). In the growth of the total biosphere carbon pool, the vegetation carbon pool provided 4.91 GtC, equivalent to 0.18 GtC per year, of which the abandonment process contributed 0.78 GtC, about 0.03 GtC per year. The soil carbon pool provided 4.32 GtC, about 0.16 GtC per year, of which 0.20 GtC was contributed by the abandonment process, about 0.01 GtC per year. Compared with the result simulated by fixed cropland, the total carbon sink is very similar to the default result because the cropland change in HYDE is underestimated, which means that the original HYDE cropland data may not express the cropland abandonment process very well in this region.

The difference between optimized and fixed cropland scenarios are 0.99 GtC, which could be treated as the increasing carbon pool due to cropland variation (the abandonment and partly re-cultivation), and within which, about 80% is contributed by the vegetation carbon pool and the remaining 20% is driven by soil carbon pool (Figure 6b,c). 

The vegetation carbon stock quantity in the RUB region changes with the variation of the forest area in phase, which means the carbon sink contributed by cropland abandonment existed in the rebirth forest vegetation. Particularly, in the process of abandoned cropland transforming to grassland/forest, although SOC from these three scenarios all increased generally, SOC from optimized croplands was lower than the other two at the beginning period until about 2004, and then were obviously larger since about 2009 (Figure 6c). 

**Figure 7:**
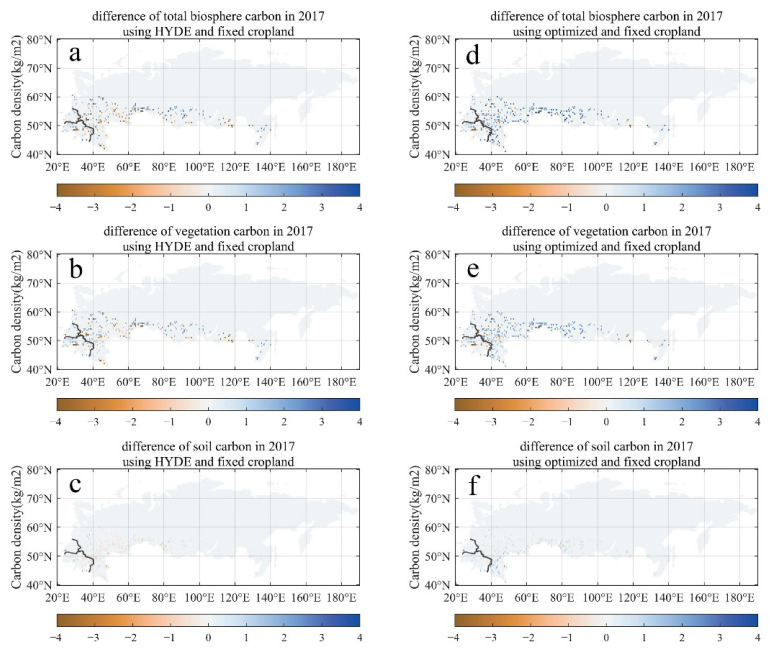
The spatial distribution of carbon pool in 2017. (**a**–**c**) the differences of the biosphere, vegetation, and soil carbon pools between HDYE and fixed cropland scenarios. (**d**–**f**) the differences of the biosphere, vegetation, and soil carbon pools between optimized and fixed cropland scenarios.

The spatial patterns of the carbon pools and their differences between optimized/HYDE cropland and fixed cropland in 2017 is illustrated in Figure 7. It can be seen that there is no significant difference between the carbon pool based on HDYE data and the fixed cropland in 1990 (Figure 7a–c). In contrast, carbon pools (biosphere, vegetation, and soil) using optimized croplands in 2017 have all demonstrated obvious general enhancements compared with the fixed cropland scenario (Figure 7d–f). The spatial patterns of the carbon pool enhancement are coherent with the extension of natural vegetation (Figure 3).

## 4. Discussion

In this study we simulated the carbon cycle of the RUB region using optimized cropland distribution. A very typical feature related to LUCC driven carbon cycle is the cropland abandonment after the Soviet Union, although the cropland area has increased slightly in recent years. Simulations have demonstrated that, using our optimized cropland data, natural vegetation expansion, vegetation carbon pools, and soil carbon pools have all changed significantly compared with both HYDE and fixed cropland scenarios. 

LUCC is closely related to social policy, especially in the context of drastic social changes across the country. Therefore, several studies have investigated cropland abandonment after the collapse of the Soviet Union. A relatively consistent conclusion from these results is that there is an increase in vegetation productivity or carbon sinks due to cropland abonnement. For instance, Vuichard et al. [34] used the process-driven ecosystem model ORCHIDEE-STICS to simulate the secondary succession of the abandoned land in Post-Soviet countries from 1991 to 2000 using FAO-based cropland records, and reported a cumulated carbon sink of 64 TgC over the domain considered, which defines a mean annual carbon sink of 0.0064 GtC. This rate is very small compared with our estimation of 0.04 GtC yr^−1^. As illustrated above, our default simulation using FAO-based HYDE data is quite similar to the simulations with fixed cropland. Therefore, cropland distributions play an essential role. 

Previous simulations by improving cropland distribution have also been carried out. Schierhorn et al. [15] estimated European Russia, Ukraine, and Belarus cropland distribution at 1 km^2^ resolution and applied the LPJmL model during 1990–2009. During this period, a net carbon sink of 470 TgC was suggested, and its annual change rate of 0.0235 GtC yr^−1^ is quite similar to our estimation. However, there is a significant difference between these two estimations. Schierhorn et al. suggested the soil carbon pool contributed much more than the vegetation carbon pool [15]. In contrast, we found the vegetation carbon pool plays the dominant role, and is four times the soil carbon pool because vegetation growth is much faster than soil organic carbon accumulations. 

The unavailability of regional-scale biomass observations makes direct verification of the simulation results infeasible. Using national-scale statistics records rather than HYDE or FAO data, our estimate and the number suggested by Schierhorn et al. [15] are consistent, and both are all much larger than the results from an earlier work by Vuichard et al. [34]. Therefore, the improvement of the input data is necessary. Another issue relies on how to verify the dominant role between vegetation and soil carbon pools during the cropland abandonment processes. The general mechanism is that, usually, abandoned croplands are converted through several successional stages to grassland and then to forest. Solid field work in Russia had suggested that after the abandonment, purely grassland stage only maintains for 2–3 years, and first-forest stage could be well established after 12–17 years [35,36]. Our simulation covers a period of 28 years. Meanwhile, this was also evidenced by the remote sensing method [22]. Therefore, rapid increases in forest re-growth and corresponding vegetation carbon pools can be expected.

Regardless of the research method used, there are huge errors in the carbon pool research in this field. The region lacks long-term field observations and systematic national censuses. For example, China has clarified the general carbon sink function of ecological engineering through censuses [37]. Meanwhile, high-quality LUCC maps are the basic prerequisite, which is usually hard to achieve. For instance, a recently released report on arable and abandoned land across former Soviet Union countries at a 10 arc-second resolution is only available for the year of 2010 [27]. Quantifying the amount of cropland that becomes grassland or forest, and the timing of the change (the age of the forest is an important parameter), is an important factor affecting carbon balance estimates. However, current annual land cover data barely reflect this information. For instance, Yue et al. [38] preferred to use MODIS C5 land cover data rather than the latest C6 version because C5 may play a better role in LUCC. Another huge error exists in soil carbon pools. It is extremely difficult to accurately simulate the accumulation and respiration of soil organic carbon, and remote sensing methods are also difficult to apply. Therefore, large-scale surveys seem to be indispensable. 

Therefore, the issue addressed here is still far from resolved, and this study is just an attempt to give a new estimate as reasonably as possible under the existing conditions. Hopefully, more independent studies in the future could narrow the uncertainties.

## 5. Conclusions

Based on the sown area data of the Russian Federation from ROSSTAT and cropland area data from FAOSTAT, we calibrated the cropland distribution of HYDE in Russia, Ukraine, and Belarus, three former Soviet grain-producing areas. The optimized croplands demonstrated that the area of abandoned croplands is significantly larger than the default data. The calibrated cropland maps were then fed into the dynamic vegetation model VEGAS to simulate the impact of this process on the ecology and carbon cycle process, evaluate the impact of the abandonment process on the carbon sink growth in this region, and evaluate the contribution of the abandonment process to the carbon sink growth in this region. The main conclusions are as follows:

(1) Between 1990 and 2017, the organic carbon sink in the RUB region increased by 9.23 GtC, of which 0.99 GtC was contributed by the cropland abandonment process. In other words, the abandoned area contributed 0.04 GtC per year to the net carbon sink growth of the area.
(2) Most of the carbon sinks contributed by the cropland abandonment process exist mostly in vegetation, where abandoned cropland is replaced by forests, which is the main factor for the growth of the vegetation carbon pool in this region. The soil carbon pool contributes a small part of the above net carbon sink, and it takes a long time for the soil to exceed the accumulation rate of the fixed scenario with no cropland change.

## Data Availability

Country-level Arable Land and Land Under Permanent Crops statistics of the Russian Federation from FAO [39] is available at http://www.fao.org/faostat/en/#country/185 (accessed on 21 October 2019); Sown Areas of Crops from the Federal State Statistics Service of Russia is at https://eng.rosstat.gov.ru/Publications (accessed on 19 January 2020); Historical Database of the Global Environment [40] is available at https://themasites.pbl.nl/tridion/en/themasites/hyde (accessed on 23 November 2020).
